# Subdural Empyema from *Streptococcus suis* Infection, South Korea

**DOI:** 10.3201/eid3003.231018

**Published:** 2024-03

**Authors:** Sejin Choi, Tae-Hwan Park, Hyun-Jeong Lee, Tae Hyoung Kim, Jin-Deok Joo, Jisoon Huh, You Nam Chung, Sang Taek Heo, Eui Tae Kim, Jong-Kook Rhim

**Affiliations:** Seoul National University Hospital, Seoul, South Korea (S. Choi, T.-H. Park);; Jeju National University College of Medicine, Jeju, South Korea (H.-J. Lee, T.H. Kim, J.D. Joo, J. Huh, Y.N. Chung, S.T. Heo, E.T. Kim, J.-K. Rhim);; Jeju National University Graduate School, Jeju (T.H. Kim);; Jeju National University Core Research Institute, Jeju (E.T. Kim)

**Keywords:** subdural empyema, *Streptococcus suis*, bacteria, raw pork, South Korea, foodborne infections, food safety

## Abstract

In Jeju Island, South Korea, a patient who consumed raw pig products had subdural empyema, which led to meningitis, sepsis, and status epilepticus. We identified *Streptococcus suis* from blood and the subdural empyema. This case illustrates the importance of considering dietary habits in similar clinical assessments to prevent misdiagnosis.

*Streptococcus suis* is a zoonotic pathogen that affects pigs and humans when they handle pigs or eat undercooked pork products. Globally, an outbreak of infection occurred in China in 2005, and *S. suis* is a common cause of bacterial meningitis in Vietnam and Hong Kong (*1*,*2*). High-risk eating habits of ingesting raw or undercooked pork also have been reported in Thailand (*2*).

Although *S. suis* infection traditionally is associated with pig contact or consumption of undercooked pork, South Korea reported its first human infection in 2012, with subsequent cases not explicitly linked to pigs (*3*). Of note, consuming raw pork is rare in South Korea because of cultural taboos. In South Korea, the prevalence of *S. suis* infection was 12.6% among slaughtered pigs and 16.4% among diseased pigs; serotypes 2 and 14 were predominant in the Jeju area compared with other regions (*4*).

Common manifestations of *S. suis* infection are meningitis, endocarditis, septicemia, and arthritis but not subdural empyema (*2*). Subdural empyema is a rare but serious infection that causes a collection of pus between the dura and arachnoid layers of the meninges (*5*). We describe a case of subdural empyema caused by to *S. suis* infection after the consumption of raw pig products in Jeju Island, South Korea, where the pork industry has been an economic pillar for over 500 years. 

The patient, a 76-year-old man, visited the emergency department exhibiting dysarthria, neck stiffness, and right-sided weakness with motor grade III. He did not have hearing loss, a common symptom of human *S. suis* infection, or signs of increased intracranial pressure such as papilledema. His medical history included a fall 3 months prior and recent headache and dizziness. Initial brain computed tomography and magnetic resonance imaging showed chronic subdural hematoma (cSDH) with recurrent bleeding and an inflamed subdural sac (Figure 1). Concurrently, he exhibited septic symptoms, such as fever, hypotension, marked thrombocytopenia, and elevated inflammatory markers, necessitating immediate administration of antibiotics (vancomycin, ceftazidime, and metronidazole). Further studies showed that he did not have endocarditis, sinusitis, or otitis media (all possible causes of subdural empyema) (*5*). We drew blood cultures on admission day and on hospital days 4 and 7 and incubated them for >5 days. On hospital day 4, we detected *S. suis* from a blood culture. Subsequent inquiries into the patient’s dietary habits revealed recent consumption of Ae-Jeo-Hoe, a traditional dish from Jeju Island, made by slicing open the belly of a pregnant pig, finely chopping or grinding the fetus, and eating it raw with various seasonings. Consequently, we conducted further microbiologic investigations to rule out other conditions, such as severe fever with thrombocytopenia syndrome and cysticercosis, which all turned out negative.

**Figure 1:**
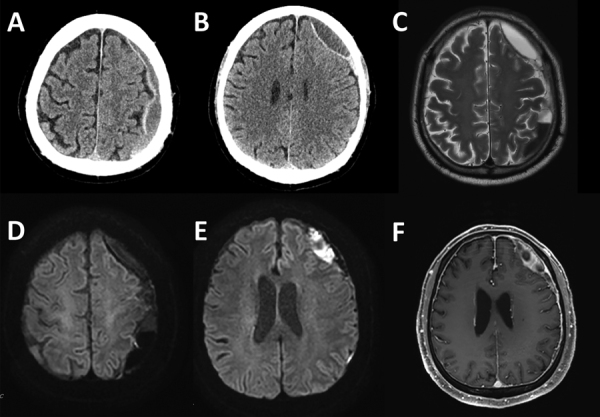
Initial image findings of subdural empyema in a patient with *Streptococcus suis* infection, Jeju Island, South Korea. A, B) Computed tomography scans. D–F) Magnetic resonance imaging: diffusion weighted (D, E), T2 (C), and enhanced T1 (F).

Upon confirmation of *S. suis* infection, treatment shifted to ceftriaxone. Results of blood cultures from days 4 and 7 were negative, but neurologic deficits persisted. On day 10, we evacuated a subdural empyema through left frontal and parietal burr hole trephinations. Intraoperatively, we identified a multiseptated pus-like tissue and a bloody subdural fluid. Despite the negative swab and fluid culture results, PCR confirmed *S. suis gdh* and *thrA* genes in the subdural empyema sample (Figure 2). We extracted total genomic DNA from blood-cultured bacteria and from the patient’s subdural hematoma by using the Solg Genomic DNA Prep Kit (SolGent, http://www.solgent.com) according to the manufacturer's instructions. We detected *S. suis* DNA by using the *gdh*-specific primers GCAGCGTATTCTGTCAAACG (forward) and CCATGGACAGATAAAGATGG (reverse) (*6*), and the *thrA*-specific primers GAAAATATGAAGAGCCATGTCG (forward) and GACAACGAACATAACAGAAACTTC (reverse) (*7*). In addition, we conducted next-generation whole-genome sequencing (Theragen Bio, https://www.theragenbio.com), which identified the isolate as serotype 2, which closely matched the genetic sequence of the ISU2614 strain (GenBank accession no. ASM1348816v1), known for its high virulence (*8*,*9*) (Appendix Figure).

**Figure 2:**
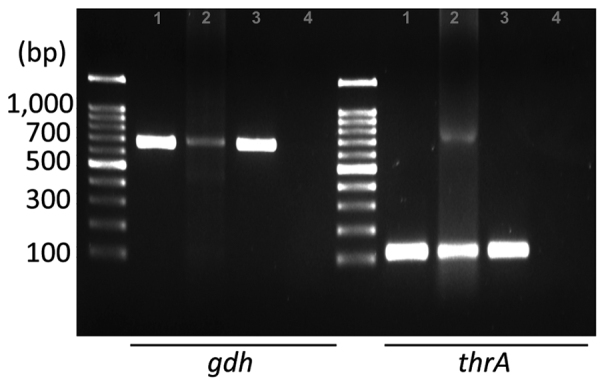
Detection of *Streptococcus suis* in a patient with *Streptococcus suis* infection, Jeju Island, South Korea, performed by using PCR with specific primers for *gdh* and *thrA*. Size marker, 1 kb DNA ladder (LugenSci, https://www.lugensci.com). Lane 1, blood culture, DNA from patient’s blood culture; lane 2, subdural empyema, DNA from patient’s subdural pus; lane 3, positive control, DNA from previously isolated *S. suis* stock; lane 4, negative control, no template PCR condition.

On day 16, the patient had onset of convulsive status epilepticus potentially attributable to meningitis, which was managed by a continuous infusion of propofol. The patient was subsequently stabilized, and his neurologic symptoms improved.

This case is noteworthy because it represents a neurosurgical condition, specifically subdural empyema, which required surgery, associated with *S. suis* infection. In addition, severe conditions such as sepsis and status epilepticus after the infection underscore the lethality of this zoonotic pathogen.

The role of the patient’s cSDH caused by prior trauma warrants further discussion in the pathogenesis of the infection. The vascularized membrane of the cSDH may have served as a seeding bed for the hematogenous spread of the infection and development of the subdural empyema (*10*), suggesting increased susceptibility in patients with cSDH or older patients with a trauma history and emphasizing the need for prompt diagnosis and treatment.

This report highlights a unique correlation to consuming a traditional dish prepared from raw pig fetuses and underscores the importance of considering dietary habits in the clinical assessment. It also raises public health concerns about the potential risks associated with consuming raw or undercooked pork products and possible *S. suis* endemic in Jeju Island, where extensive pig rearing and consumption take place. Increasing disease awareness among clinicians and laboratories can prevent undiagnosed or misdiagnosed cases.

Appendix Additional information about subdural empyema from *Streptococcus suis* infection, South Korea.
