# Supplementary material for: Subdural Empyema from Streptococcus suis Infection, South Korea
Source: Emerg Infect Dis. 2024 Mar;30(3):616–9. doi: 10.3201/eid3003.231018 (PMC10902535; doi:10.3201/eid3003.231018)
Supplement: Appendix — Additional information about subdural empyema from Streptococcus suis infection, South Korea. [file 23-1018-Techapp-s1.pdf]

Article DOI: https://doi.org/10.3201/eid3003.231018

Subdural Empyema from *Streptococcus suis* Infection, South Korea

Appendix

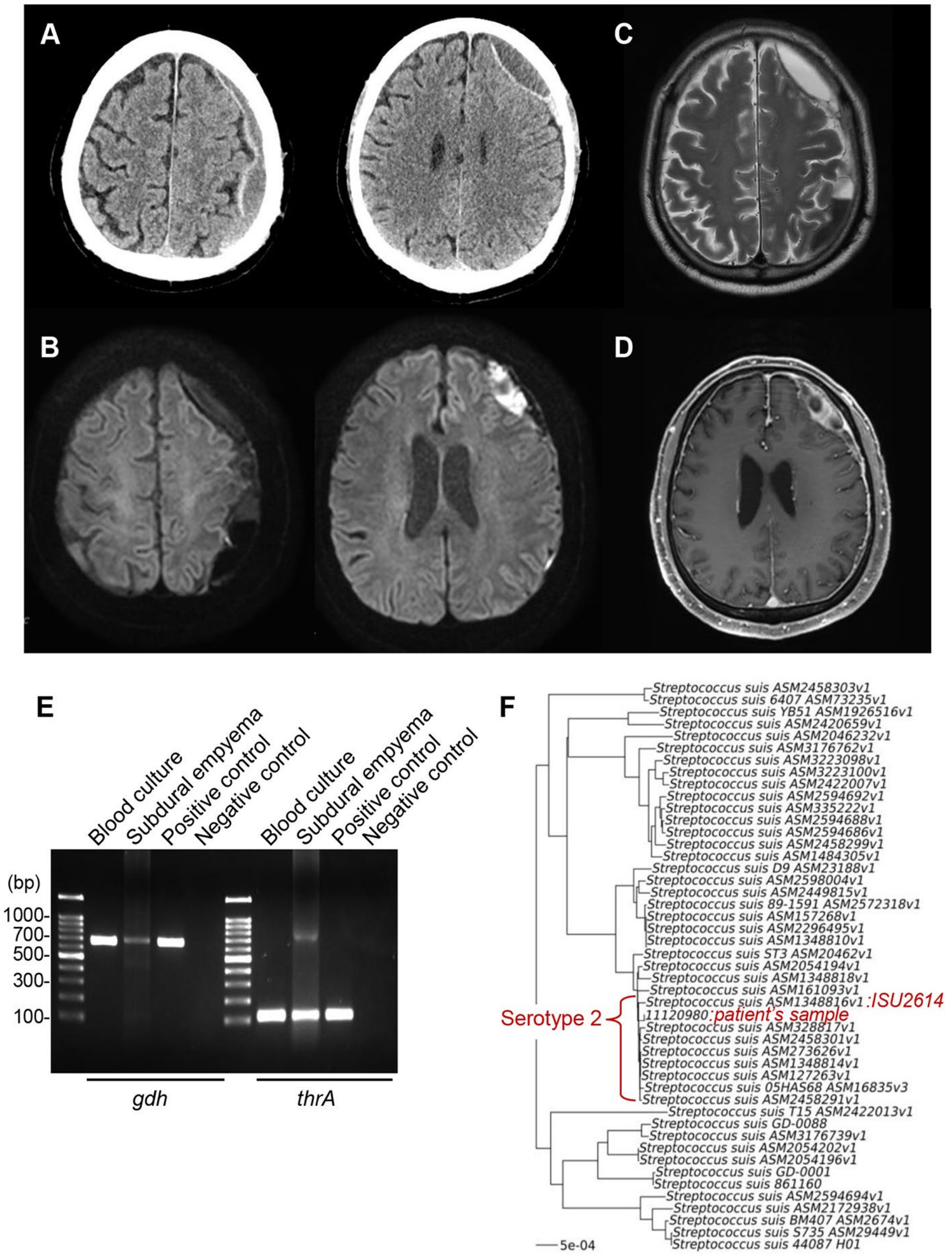

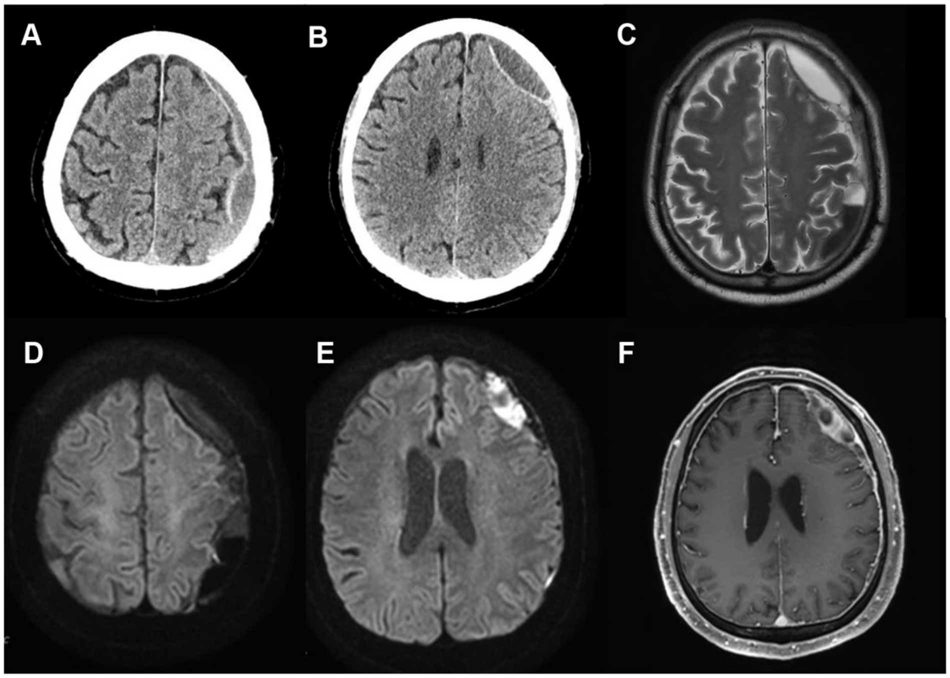

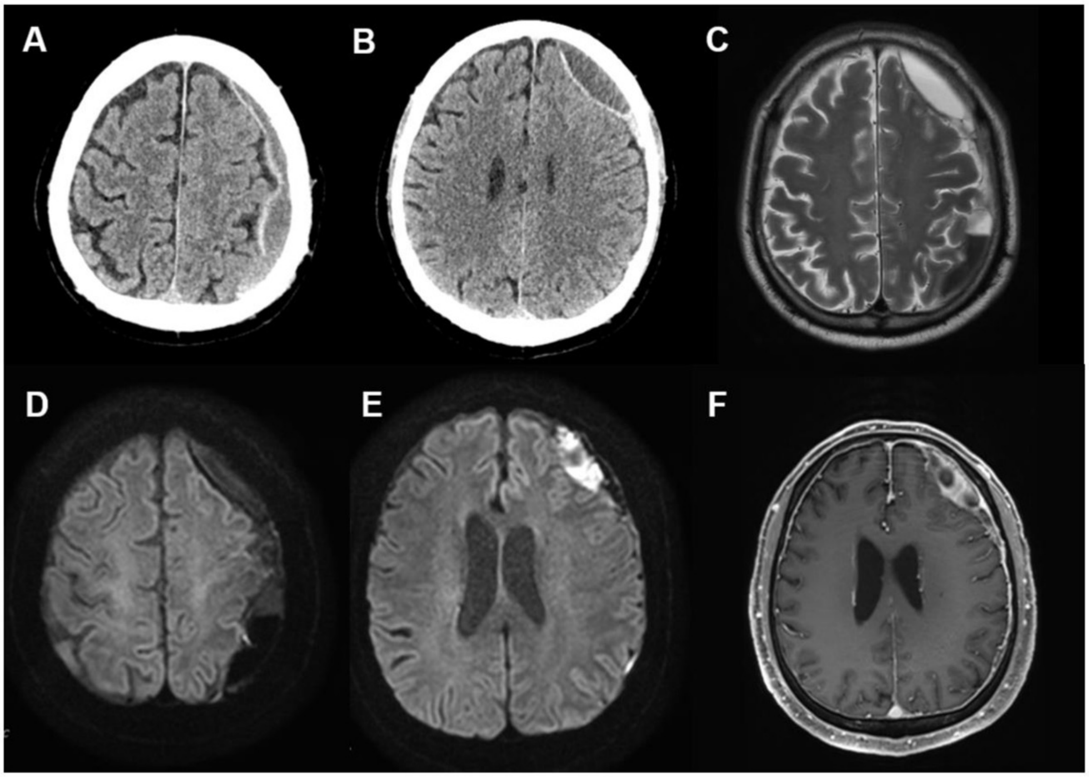

Appendix Figure. Phylogenomic tree of blood cultured *Streptococcus suis* from the patient and related type strains. Whole-genome phylogenies based on a maximum-likelihood (phylogenomic) tree inferred from 49 genomes. Scalebar indicates 0.0005 substitutions per nucleotide position.
